# The Microbiome of the Built Environment: The Nexus for Urban Regeneration for the Cities of Tomorrow

**DOI:** 10.3390/microorganisms10122311

**Published:** 2022-11-22

**Authors:** Antonia Bruno, Sara Fumagalli, Giulia Ghisleni, Massimo Labra

**Affiliations:** Biotechnology and Biosciences Department, University of Milano-Bicocca, 20126 Milan, Italy

**Keywords:** sustainability, smart cities, urban, built environment, microbiome, hygiene, bioinformed design, MIGI, hospital microbiome project, MetaSUB

## Abstract

Built environments are, for most of us, our natural habitat. In the last 50 years, the built-up area has more than doubled, with a massive biodiversity loss. The undeniable benefits of a city providing all the basic needs to a growing population showed longer-term and less obvious costs to human health: autoimmune and non-communicable diseases, as well as antimicrobial resistance, have reached unprecedented and alarming levels. Humans coevolved with microbes, and this long-lasting alliance is affected by the loss of connection with natural environments, misuse of antibiotics, and highly sanitized environments. Our aim is to direct the focus onto the microbial communities harbored by the built environments we live in. They represent the nexus for urban regeneration, which starts from a healthy environment. Planning a city means considering, in a two-fold way, the ecosystem health and the multidimensional aspects of wellbeing, including social, cultural, and aesthetic values. The significance of this perspective is inspiring guidelines and strategies for the urban regeneration of the cities of tomorrow, exploiting the invaluable role of microbial biodiversity and the ecosystem services that it could provide to create the robust scientific knowledge that is necessary for a bioinformed design of buildings and cities for healthy and sustainable living.

## 1. Introduction

The world is becoming more urban every day: only 40% of the global built-up environment (updated to 2016) was constructed before 1975, implying a more than double expansion of built-up surfaces [1]. Along with urbanization, another main protagonist of the last decades has been population growth. It is estimated that from 2020 to 2050, the percentage of the global population living in urban areas will increase from 55% to 67% [2]. Cities are indeed growing by size and by number: by 2030, 706 cities are expected to have at least 1 million residents [3]. The high-density transformation of urban areas is due to the higher living standards that urban populations experience. They have access to elevated sanitation services, to easily available drinking water, electricity, and clean fuels, and malnutrition rates are lower [2]. However, at the same time, we are recording an escalation in non-communicable chronic diseases and autoimmune diseases [4,5]. There is increasing evidence that an inadequate exposure to microbial diversity from early life to old age plays a role; industrial advances include antibiotics, processed food diets, and a highly sanitized environment, and such conditions have been shown to influence microbiota composition and transmission [6,7,8,9]. The undeniable benefits of a city providing all the basic needs to a growing population have shown longer-term and less obvious costs to human health.

**Figure 1:**
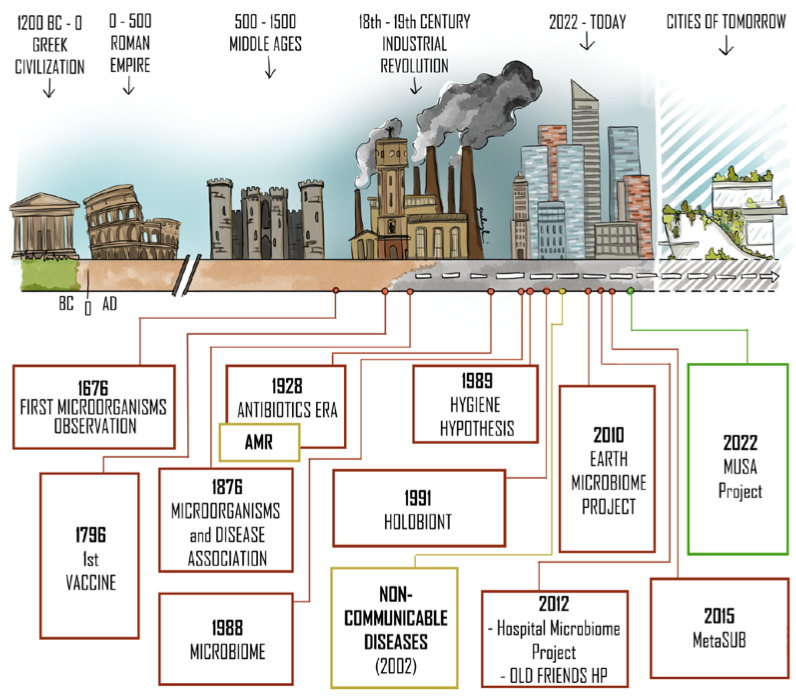
Urbanization timeline and scientific milestones roadmap. This schematic and not exhaustive representation covers the main milestones of urbanization (in the upper part) and the key scientific findings and projects that allowed us to gain awareness about the microbiome of the built environments (in the lower part). From ancient civilizations to nowadays, urbanization has increased enormously. Despite cities and inhabited areas having developed for thousands of years, only recent scientific advances have enabled the evaluation of the influence of urbanization on the microbiome of the built environment (BE) and human health. The end of the roadmap is projected towards the cities of tomorrow, and, in this context, the MUSA (Multilayered Urban Sustainability Action) project [29] was born. AMR: antimicrobial resistance; OLD FRIENDS HP: Old Friends Hypothesis; MetaSUB: The Metagenomics and Metadesign of the Subways and Urban Biomes.

Indeed, the microbiota harbored by individuals living in the industrialized world is of a configuration never before experienced by human populations, with a significant loss in biodiversity and an increasing vulnerability [10,11,12,13]. Before the first observation of microorganisms by Antoni van Leeuwenhoek in 1676, the presence of the microscopic “city-symbionts” were unimagined [14,15]. Some therapeutic treatments (such as the first smallpox vaccine by Jenner in 1796 [16]) were indeed idealized well before the demonstration of the correlation between some microorganisms and certain diseases (Koch, 1876 [17]). Following this key progress, and coinciding with the penicillin discovery by Fleming in 1928 [18], the “Antibiotics Era” began. Although antibiotics notably improved the terrible health conditions inherited from the cities of the industrial revolution, the inevitable evolutionary mechanism of selective pressure began to shape what has now become a major health threat: antimicrobial resistance (AMR) [19]. More recently, the study of the interaction between humans and microorganisms gained attention. The concept of the microbiome was indeed first defined in 1988 by Whipps et al. [20]. During the subsequent years, the Hygiene Hypothesis (1989) [21] and the concept of the “Holobiont” (1991) [22] were formulated (as discussed in Section 3). Together with the Old Friends Hypothesis (2012) [23], these theories enabled a novel insight into Non-Communicable Diseases (NCDs) that heavily affects modern citizens and that is derived from our modern lifestyle. WHO reports that 74% of global deaths are caused by NCDs [24], thus, major changes to improve life conditions and their health implications are imperative. To meet these needs, numerous microbiome-centered projects were born, such as the Earth Microbiome Project (2010) [25,26], the Hospital Microbiome Project (2012) [27], and MetaSUB (2015) [28]. The two-tier progression of urbanization and scientific innovations should converge in a new approach for the cities of tomorrow (Figure 1).

The question we want to bring to the forefront, and that represents one of the “dark sides” of living in urbanized areas, is how does the urban environment impact human health? New technological advances in molecular biology (i.e., metagenomic and in general omics approaches) are opening up new possibilities in investigating the nexus between human health and ecosystem health, shedding light on the role of the host-associated and environmental-associated microorganisms. 

The microbiome of the built environment (BE) refers to the microbial communities harbored by the environments that humans have constructed, including houses, offices, public buildings (such as schools), cars, roads, and public transport, but also drinking water treatment plants and other human-built spaces [30,31,32,33,34,35,36].

In recent years, substantial research into the presence, abundance, and diversity of microorganisms in the built environment (BE) has taken place. However, the notable review of Li and colleagues about the built environments, occupants, and microbiomes [37] pointed out the general lack of research effort linking built environment attributes with the microbial communities harbored by the built environment itself. 

The time is ripe for a bioinformed design [38], where the incorporation of expertise from architecture, engineering, public health, microbiology, and ecology allows both confident and interdisciplinary analyses, creating the robust scientific knowledge that now is urgent for sustainable living. In this context, the MUSA (Multilayered Urban Sustainability Action) [29] project has been conceived to lead the transition of a metropolis (such as Milan, Italy) towards environmental, economic, and social sustainability. It promotes a science-based approach to the multidisciplinary engagement of citizens, scientists, industries, and public administrations, reflecting an unprecedented effort for urban ecosystem health.

**Figure 2:**
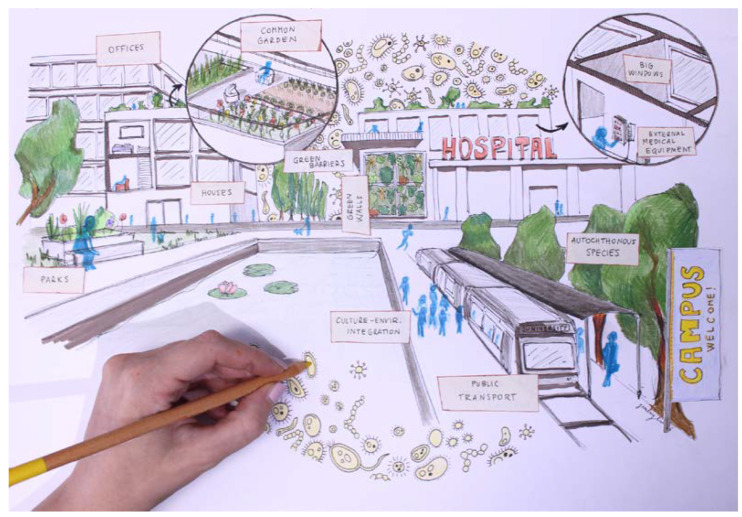
The new “Urban”: Projects regarding future everyday life hot-spots is something that must be done today; in the illustration, a hand is indeed drawing the vision for a new meaning of infrastructure. The key themes are functions integration and social inclusion through bioinformed design and architecture. The same spaces have multiple functions. The park becomes a recreational (but also educational) area of a university campus underlying the imperative role of knowledge in re-modelling the environment. Public transportation lines are surrounded by trees, vegetation, and natural elements such as ponds and gardens, so that autochthonous species become reintegrated. Houses and workplaces are designed to be well-lit environments, with direct access to nature thanks to the presence of terraces and common gardens (that also favor socializing and manual activities such as gardening and horticulture). Architectural elements, such as acoustic barriers and structural walls, are implemented along with green-promoting elements. Lastly, hospitals are re-designed to promote physical and mental health via lighter and greener accessible areas, on one hand, and via the reduction in the transmission of possible pathogenic microorganisms (isolating patients from commonly touched medical equipment). The microbiota is represented as the human–health–environment linking element.

Our argument is that a new impact- and purpose-oriented environmental model, coupled with appropriate scientific tools, may offer a huge opportunity to reconcile and balance the growth of modern societies. To achieve this goal, we have to unleash the potential of sustainable approaches by leveraging the hidden virtues of biotechnological innovation. Molecular technologies combined with environmental sensors can be used to assess and improve ecosystem support services (e.g., air and water purification, soil regeneration), regulation (contrasting climate change, promote pollination and seed dispersal), and cultural value (e.g., shared education, aesthetic appreciation, recreation improvement) in urban contexts. Predictive models (i.e., microbial co-occurrence analysis and machine learning approaches) and remote sensing systems can be used to (i) predict equilibrium perturbation in order to adopt preventive measures and to (ii) promote biotechnological exploitation to improve the quality and safety of the urban ecosystem. Data generated should be made available in dedicated repositories following all the applicable best practices for making data FAIR (Findable, Accessible, Interoperable, and Reusable) [39]. In this context, cities can be seen as living labs, with campuses at the core. Through this vision, the involvement of citizens and students is crucial: citizens and students can have a role in the collection of Big Data for the co-production of health. In this way, models of social participation through a Responsible Research Innovation (RRI) approach will nourish the design of new neighborhoods (Figure 2).

The aim of this perspective is to move the focus to the microbial communities harbored by the built environments we live in, inspiring guidelines and strategies for the urban regeneration of the cities of tomorrow.

Starting from a lesson from the past, when the issue of built environments in health and disease started to rise, we will cover the main topics about the crucial role of microorganisms (by placing the emphasis on bacteria) and their interaction with us and the built environments. Then, we will discuss Microbiome-Inspired Green Infrastructure (MIGI) as a possible way to counterbalance urban dysbiosis and to promote a bioinformed design. The emblematic case of the MetaSUB Consortium is proposed as an example of a coordinated research effort to build the robust scientific knowledge that is necessary for the bioinformed design of buildings and cities. Finally, a specific focus on an extreme ecosystem, such as a hospital’s built environment, will address the main concerns of the biodiversity loss and spread of multidrug resistance.

## 2. Lessons from the Past about the Role of the Built Environments in Health and Disease

Ancient Mediterranean societies had a developed understanding of the importance of building design and architecture for public health. Not only public buildings but the cities, as well, were conceived to minimize stagnant air and humidity. In the Hippocratic Corpus (dating back to the 5th–4th century BC), we can find one of the first analyses of airflow and humidity and its influence on seasonal peaks of infectious diseases [40]. The Roman architect and engineer Vitruvius (1st century BC), without having knowledge about microorganisms and the real causes of infectious diseases, advised building cities on an elevated point and far from swamps and mosquitoes. Similarly, when he described how a theater (a public building) should be, he linked the structure of the building to the airflow and, thus, to the health of the occupants [41].

In modern societies, prior to the development and adoption of antibiotics and vaccines, often, the treatment of some diseases was environmental. A clear example was the sanatorium movement [42], born in Europe and the United States in the late 1800s: before the development of medications for tuberculosis, sanatoria were spaces designed to house, isolate, and treat patients. Hygiene and ample exposure to air and sunlight were their strong suits. These new clinical environments anticipate a new modern architecture that has been reflected in the work of the Swiss architect Le Corbusier. In “The City of Tomorrow and Its Planning” (1929) [43], he pointed out the inadequate housing and inefficient transportation that grew out of the unplanned jumble of medieval cities. Although some aspects of his vision can be criticized, he contributed to reinforcing awareness in a new way to imagine cities, where indoor spaces are full of sunlight and clean surfaces and outdoor spaces are planned based on function. Terraces, balconies, and flat roofs are now common elements in modernist architecture, as well as a new sensitivity to materials used in buildings. Beyond their aesthetic appeal, these features embodied modernist preoccupations with the healing effects of light, air, and nature (Box 1). 

Nowadays, the research focused on the microbiome of the built environment can make a significant contribution to the safety, resilience, and survival of our ecosystem and health. Its exploitation can address the call of SDG 11 for sustainable cities and communities [44,45].

Box 1 **The old new role of balconies.**

Balconies are an ancient architectural archetype, but nowadays, they are rela-tively scarce in many of the densest urban areas and represent one of the top ameni-ties, especially after the pandemic’s social restrictions [46]. Indeed, they are considered to improve the home’s livability, the building’s architectural interest, and the proper-ty’s green performance. On that last point, balconies can help lower energy use by providing “passive shade” that can naturally cool down homes. According to a recent study [47], balconies produce relevant impacts in four factors that contribute to indoor environmental quality: thermal comfort, indoor air quality, visual comfort, and acous-tic comfort.

In our urbanized societies, living in natural environments is slowly slipping away, but balconies, as well as rooftops, terraces, or courtyards, can provide a source of semi-natural biodiversity. Being a natural connection with the outdoors and often decorated with plants, they can contribute to the biodiversity of a house, with benefits for human health: a recent study demonstrated that green walls positively affect skin microbiome diversity and reduce proinflammatory cytokines of the occupants [48]. Moreover, in green environments, the air contains not only variable microbial species but also biogenic chemicals, including volatile organic compounds (VOCs) such as limonenes and terpenes, which can be anti-inflammatory, antioxidative, and anxiolyt-ic [49]. Therefore, balconies can be a good compromise to bring nature back to a built environment.

## 3. A Long-Lasting Alliance with Microbes

Like it or not, we are living in a microbial world, to twist the lyrics of a famous Madonna song and, also, cite a growing number of scientific papers and books [50,51,52,53,54]. This awareness is not new to science. Lourens Gerhard Marinus Baas Becking (1895–1963) is known for the Baas Becking hypothesis: “Everything is everywhere, but the environment selects”. Baas Becking’s idea was that while all microbial life is distributed worldwide, specific microorganisms are observed in specific environments, with their own peculiar characteristics [55].

We are currently experiencing the slow transition from fear to reluctant acceptance of the microbial world [31], while entering what Glenn A. Albrecht defined the “Symbiocene” [56]: the next human era that will revolve around the importance of the interconnectedness of all living things.

Microbes are part of a wide and deep ecosystem service, contributing to human health and, at the same time, the well-being of the Earth itself.

Indeed, humans can be viewed as holobionts (where “holos” means all, whole, a definition belonging to Lynn Margulis: Margulis and Fester, 1991 [22]), “mega-organisms” composed of the host and the interacting viruses, bacteria, and other (micro)organisms, which together form a discrete ecological unit. This collection of microorganisms, the microbiota, is not merely a random assembly of microbes emerging from the environment and/or selected by chance; rather, specific host–microbiota interactions are maintained over time by selection and, together, they (we) coevolved, developing tight relationships. We could indeed say that the host’s physiology depends not only on its genome but also on its interactions with all the genomes of the microbiota: the microbiome. A remarkable example is represented by ruminants: the digestive ability exclusively present in some microorganisms extended the food-source range of these host animals, resulting in the positive selection for this advantageous trait and in the long-term radiation of this taxa [53]. We can compare the selection of a stable interaction to the evolution of a novel genetic trait. Going back over a billion years in time, we find the most affecting result of bacterial endosymbiosis: the origin of mitochondria and plastids. As Wein and colleagues [57] argue, a stable symbiosis is established when the two counterparts exchange a certain currency (photoautotrophy in the case of plastids, and energy preservation in relation to mitochondria), reaching a mutually beneficial trade. In addition, to identify an evolutionarily selected interaction, the mechanisms of currency exchange (transport proteins for the mentioned organelles) and the means of inheritance of the interaction over generations (organelles segregation mechanisms) have to be defined.

Shifting the focus to human beings, the ancient and long-lasting alliance between hosts and their microbiota contributed to shaping our capability to adapt to the world of the past [5]. Facing the nowadays expeditious changes in human lifestyle and environment, evolutionary mismatches between the “outdated” currency exchange mechanisms and the anthropogenic environment give rise to novel diseases of civilization [58]. Thus, the role of the environment (with its microorganisms) appears crucial in the equilibrium of this interaction. A significant increase in inflammatory-related diseases’ frequency has characterized urbanized populations over the last decades, and a proportion of this rise (with regards to pathologies such as asthma and allergies) seems to be dependent on the evolutive consequences of the lack of exposure to immuno-regulative microorganisms. This principle is described by the Hygiene Hypothesis, and it contributed to the development of a new conception of “hygiene”. “Hygiene” has been a central daily life topic during these several last years, spent learning to cope with the pandemic spread of SARS-CoV-2. We learned to wear gloves in grocery stores, to periodically disinfect our hands, and to dress in any sort of protective equipment in order to isolate ourselves from the surroundings. Although this was the only powerful strategy that slowed down the spread of the virus when vaccines were not available, this attitude is in complete contrast with the change in the microorganisms-related ideology sponsored by the biology of the last decades. Removing all microorganisms is indeed very different from removing the pathogenic ones, and even though it may sound a bit counterintuitive, it is not the most beneficial or “hygienic” approach. The Old Friends Hypothesis formulated by Rook [23] defines the importance of an early childhood exposure to microorganisms as a key step in modulating the immune system and in building up a diverse and beneficial microbiota. Numerous studies have been conducted to elucidate the correlation between the changes in microbiome composition and COVID-19 severity, but the lack of comprehensive data still makes it difficult to address this relationship [59]. The next future perspective will be to use microbiota analysis as a prevision indicator of disease progression and the monitoring of its composition as a preventive measure. Even though more investigation is needed under a microbiome point of view, among the various studies driven by the pandemic’s consequences, the importance of the mismatch between the evolutive adaptation and the constantly changing environment has emerged once again. It turned out that some genetic variants linked with a high susceptibility to severe SARS-CoV-2 infection derive from an adaptive introgression of advantageous traits, subsequent to anatomically modern humans’ admixture with Neanderthals [60]. What was beneficial against ancient pathogens has become a limitation in facing environmental challenges of the present. The unstoppable and inevitable adaptation of biological systems to the anthropogenic environment indeed has a cost in terms of newly formed diseases and increased susceptibility. Therefore, health protection should, undoubtedly, be extended beyond the comprehension and the cure of these pathologies of civilization to include the design and the establishment of a salubrious BE.

## 4. Microbiome-Inspired Green Infrastructure (MIGI) to Counterbalance Urban Dysbiosis

**Figure 3:**
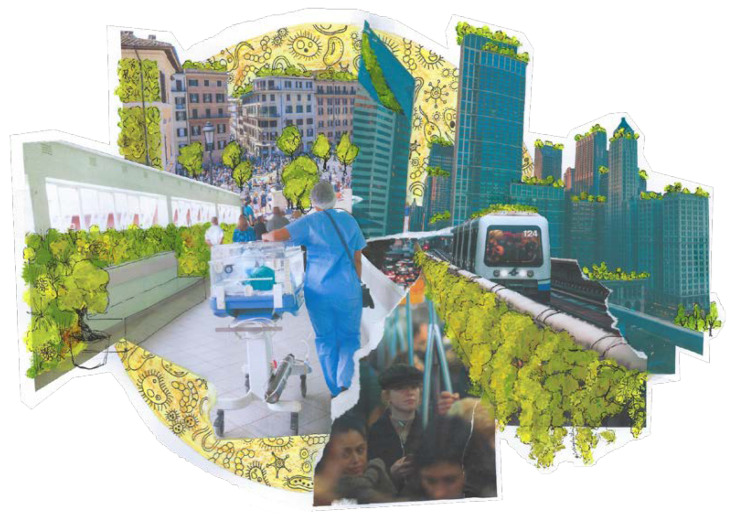
Rewilding today’s urban centers: While designing policies to address the construction of the integrative cities of the future, we will have to transition through an intermediate phase of nature’s inclusion in existing urban areas. This illustration is realized with clippings representing the city of today (a crowded square, clusters of skyscrapers, commuter subway lines, and the core of last year’s public health: hospitals). The figure’s layered structure represents the different tiers we must act on to renovate the current urbanization condition. On top of everything, painted vegetation is inserted in the already existing urban pattern: a call for greener living spaces is becoming increasingly essential. The microbiota (both human and environmental), represented in yellow in the background, envelops everything as the linking element between humans and the context we built and live in. Not only does our health depend on our microbiome, but its composition is heavily affected by environmental changes; just as in a collage, every piece is glued to the others.

To reverse the deleterious effects that a rapid and destructive urbanization has on the environment–human–microbiota ecological relationship, a renovation of the urban physical space is necessary. To promote public health and facilitate the interaction with important environmental microbiota components, the model of Microbiome-Inspired Green Infrastructure (MIGI) has been proposed [61]. The establishment of such infrastructures as part of the basic toolkit for the planning, management, restoration, and design of urban and inhabited areas will compensate for the fragility inferred on microbial networks by human intrusive actions. It requires the multidisciplinary contribution of different professional (and non-professional) figures, so that spaces are re-designed to include vegetal, animal, and microorganism species that could increase the urban ecosystem’s benefit to human immune modulation [62]. In this sense, green jobs encompass a central role, envisaging the transition to a resource-efficient sustainable and inclusive social model [63]. Urban regeneration indeed requires, by definition, cooperative public, private, and community efforts to improve quality of life for all [64], including social well-being and inclusiveness, cultural valorization, and environmental and economical sustainability among its diversified goals [65]. Microbiology has started to be integrated with other related practices, since the exploitation of microorganisms as bioremediators has been a convenient strategy to manage and decrease chemical and metal pollutants from the environment. For instance, Constructed Wetlands (CWs) are taking hold in domestic and industrial wastewater treatment technologies as a promising microbiologically-based strategy. The bacterial biofilm associated with the roots of these vegetation rafts is indeed composed by sulfate-reducing, denitrifying, nitrogen-fixating, ammonia-oxidizing, polyethylene-degrading, methanotrophic, and methylotrophic bacteria (among which Proteobacteria and Cyanobacteria are the main identified taxa) that can remove heavy metals and degrade organic contaminants [66]. More particularly, species such as *A. platensis* (commonly known as Spirulina), that belong to the Cyanobacteria, have been tested and confirmed as water bioremediators which, through the uptake of the heavy metals of these microorganisms, restrain the concentrations of toxic, nonbiodegradable, and bioaccumulative metals such as nickel [67]. Microbiology also comes to the rescue in other urban and human waste-related processes: the anthropogenic gasses (methane, above all) generated by the decomposition of landfill organic material indeed perturb the stability of the environment and the climate. To counterbalance this problem, methanotrophic bacteria are used in landfill biocovers to degrade in situ, and thus contain, the emissions of methane that would otherwise persist in the atmosphere and contribute to climate change [68]. Despite these examples clearly showing the potential of a multidisciplinary approach to sustainable urban management, in which biology has a considerable role, the practical and operational functioning of architecture make the inclusion of microbiology in landscape design more challenging. This being said, the future perspective we envision holds a big role for metagenomics and metatranscriptomics in planning a health-oriented urban renovation. The establishment of biodiverse green urban areas could indeed promote health, increasing microorganisms–human interaction and, thus, conveying diversification to the dynamic proportion of our microbiota [61]. Not only urban parks but also green barriers and roofs, rain gardens, hedgerows, wildflower verges, wildlife overpasses, and community allotments can be integrated in schools and other public buildings. With precise and mindful planning, the reintroduction of autochthonous plants can be a positive side effect, able to restore a neglected biodiversity. As a natural reservoir of microorganisms producing immunoregulatory molecules, a greener life context would face the dysbiosis caused by our urban and social sphere (both in and around humans), rewilding and, thus, introducing functionally diverse microbial species [61,69]. According to the Rewilding Hypothesis, the biodiversity of microorganisms associated with natural environments can promote the restoration of the ecosystem service of immune protection, provided by the coevolution of these microorganisms with humans [70]. In some contexts, we must preserve the value and the cultural heritage of historical sites. In other cases, we have to cope with already existing structures and buildings (Figure 3). Through a precise and reasoned way to promote nature’s inclusion in existing urban areas, i.e., urban renovation, we can design policies to modulate the construction of the integrative cities of the future.

Changing mindsets is possible at the local, but also at the national, level. A successful example is represented by the Finnish Allergy Programme 2008–2018 [71], a society-wide proactive program aiding to reduce the burden of allergic diseases and asthma. Starting from the biodiversity hypothesis of health [72], the traditional strategy was changed from avoidance to tolerance: allergy health and contacts with the natural environment were emphasized to promote immunological, psychological, and societal resilience. By improving prevention and care, promoting nature exposure and greener cities, they were able to reduce costs both at the societal and at the patient level [73]. 

Assuring parity in the reachability of high-quality green areas is the condition for an effective turning point in public health, and the transition to the common sensitization about the essentiality of the microbiome functions should concern everyone, without considering their socio-economic status, cultural background, age, sex, or residential area. In addition to the abovementioned point, we think that the major obstacle this conceptual and operational revolution has to face is the need for a holistic change: alterations must regard the roots of science and building processes, together with the cultural and educational perception of the built environment. 

## 5. In and Out: MetaSUB Highlights the Connections between Urban Biological Systems

Transit systems are the built environment where indoors and outdoors are in continuous connection, playing a key role in the microbial dissemination network. Indeed, by connecting the suburbs with the center, urban transit systems, such as the subway and buses, repeatedly open their doors to carry tourists and locals of different economic and social backgrounds. Depending on the time of day and route, passengers on board share a relatively confined space with a high propensity for microbial exposure and transmission between people and with touched surfaces [74].

MetaSUB (Metagenomics and Metadesign of the Subways and Urban Biomes, http://metasub.org/ (accessed on 10 October 2022)), funded in 2015, aims to map metagenomes of the cities of the world, focusing on mass-transit systems [28,75]. MetaSUB’s goals include both the cognitive purpose of the urban biological systems and an applicative one, with the search for new drugs and antibiotics to be used in pharmaceutical design [28]. Together with researchers and citizen scientists, MetaSUB has collected BE samples from almost all continents, starting from the iconic Global City Sampling Day in 2016 (http://metasub.org/ (accessed on 10 October 2022)). Built on FAIR principles [39], MetaSUB has striven to standardize processes, from sampling to bioinformatic analysis [28]. In addition, MetaSUB has partnered with the Critical Assessment of Massive Data Analysis (CAMDA, http://www.camda.info/ (accessed on 10 October 2022)), releasing a subset of the MetaSUB data to the CAMDA community, as an annual challenge, since 2017 [76]. As a result of MetaSUB’s and CAMDA’s community efforts, we have a wide picture of what happens in subways. Researchers have identified specific features of microbiomes from diverse subways worldwide, including diurnal variation [77], microbial differences on diverse materials [78], and pathogen transmission potentiality [79]. As expected, the most prevalent microbial communities on subway surfaces are soil- and skin-associated microorganisms due to passengers’ shoes and hands, such as *Pseudomonas*, *Brevundimonas*, and *Stenotrophomonas* [79,80]. These genera seem to have successfully evolved to live in subway conditions, including few carbon sources and the constant introduction of new microorganisms [80]. Interestingly, each city has a unique bacterial profile, a “bacterial fingerprint”, that enables sample provenance prediction [81]. By machine learning techniques, we can distinguish different cities by their urban microbiome [76,82], a piece of great news for forensic purposes. 

What about passengers? As described in the review by Peimbert et al. [83] that presents a comprehensive view of subway system-related studies (linked and not linked to MetaSUB), people on board come into contact with different subway surfaces, such as poles and seats, which have been previously touched by a large number of other people. During a ride, and touching a handrail for 30 minutes, the passenger acquires diverse antibiotic resistance genes, even if the subway surfaces are treated with antimicrobial paint [84]. Then, when he/she gets off the subway, his/her hand comprises a microbiota similar to other passengers and the subway [85].

To live in a “smart city”, as the MetaSUB consortium called it [28], mass-transit systems are the key, especially if we consider the significant boost in urbanization predicted by the U.N. [86], which will probably increase the number of people relying on public transport. Therefore, an effective transport system with proper ventilation, materials that do not promote adhesion, and adequate cleaning are some of the elements that the expert team will need to consider for the subway of the future.

## 6. A Focus on the Hospital Ecosystem: An Inhospitable Environment for Many, but Not for All

Far from being as sterile as we thought, hospitals harbor a variety of microorganisms [87]. Among indoor BEs, however, hospitals represent a unique case due to their extreme conditions for microbiota life. For example, strict specific cleaning protocols, high antibiotics administration, and inoculum of new pathogens from patients are some of the strong selective pressures to which the hospital-associated microbiota is exposed. This strong anthropogenic influence leads to the domination of human-associated bacteria with a higher abundance of opportunistic pathogens and fewer potentially beneficial bacteria over hospital floors when compared to public buildings and public and private houses [88]. As a result of constant cleaning product exposure, microbial communities gain an increased ability to degrade some of their compounds [88], such as multidrug-resistant *Staphylococcus aureus* strains found with multiple copies of disinfectant resistance genes [89]. In terms of antimicrobial resistance (AMR), hospital microbiota tends to present a wide diversity of multi-drug genes, especially those involved in efflux encoding [88]. These multidrug-resistant microorganisms (MDROs) persist in hospitals [89] and can be transmitted to patients, causing hospital-acquired infections (HAIs) [90].

It is important to note, however, that among these opportunistic pathogens, a large community of microorganisms, many harmless and some even potentially beneficial, lives in hospitals [91]. These microbial communities could form a kind of “immune system”, decreasing opportunistic pathogen accumulation and persistence in hospitals [27,87]. This was one of the issues the Hospital Microbiome Project wanted to address. The Hospital Microbiome Project, which has just reached its tenth year, was a year-long work to characterize the microbial colonization of the newly constructed University of Chicago Medical Center Hospital (Chicago, IL, USA) [92]. After a hospital’s opening, surface bacterial load increases, and microbiota composition changes to mainly skin-associated genera, such as *Corynebacterium*, *Staphylococcus*, *Streptococcus*, and *Acinetobacter* [93,94]. Indeed, skin-associated microbiota is everywhere in a hospital: in common areas [93], high-touch surfaces (such as doorknobs, bed rails, and bedroom lockers) in patients’ rooms [89,93], and even in the dust over operating room floors and lamps [95], while aquatic and terrestrial environment-associated bacteria (for example, *Achromobacter*, *Elizabethkingia*, and *Serratia*) are present in the aerator and sink trap of patients’ rooms [89]. When a hospital closes, however, human-associated microorganisms’ abundances decrease, while environmental bacteria, such as Bacillaceae, Burkholderiaceae, and Rhizobiaceae, increase [96], indicating that occupancy is the major driver of hospital microbiota [93]. 

Humans are a strong determinant of the beneficial and even pathogenic microorganisms we encounter in the hospital, and the continuous interaction between staff, patients, and their families further confound the issue. The risk is that as AMRs increase, hospitals will become reservoirs of MDROs, representing a threat not only for patients and visitors but especially for fragile subjects. Environmental microbiota species (including drug-resistant microorganisms) can indeed be transferred from ward surfaces to preterm newborns’ airways. Infants born before the 28-week threshold are prone to contract HAIs, and, due to their immature immune system, they are extremely vulnerable to these infections that are often fatal [97]. The monitoring of hospital microbiota, clearly, acquires additional importance. In Europe, more than 670,000 infections every year are caused by antibiotic-resistant bacteria, resulting in about 33,000 deaths, and people with an immature or fragile immune system, such as premature newborns and the elderly, represent the most vulnerable population [98]. 

How can this major concern be addressed? Indiscriminate sterilization with antimicrobial products promotes resistome selection, and, thus, an increase in MDROs. Modulating the cleanliness interventions based on the hospital’s area is a first step [88]. In fact, although cleanrooms must be almost microorganism-free, the same does not apply to other hospital areas that may accommodate microbial communities [88]. Opening windows to let in outdoor environmental air and introducing green plants into the hospital are simple solutions to restore microbial biodiversity [88,99] and potentially hinder opportunistic human pathogen establishment [100]. Some precise interventions for microbial restoration, instead, involve the active manipulation, also called “biocontrol”, of the indoor microbiota, for example, by the application of *Bacillus* spores [88,101]. Probiotic-based sanitation of hospital surfaces reduces pathogens (bacteria, fungi, and viruses) and thus decreases HAI incidence in an eco-sustainable manner, while preventing recontamination by steadily diminishing resistance genes in the microbiome (Box 2). However, these solutions that would counter the growing HAIs need to be carefully studied before their adoption in the long term. 

Again, collaboration among project managers, medical staff, biologists, engineers, and architects is the key to design a modern, healthier hospital.

Box 2 **Active manipulation of the microbiome in cleaning practices.**

Since the beginning of the SARS-CoV-2 emergency, disinfectants and antimicrobial detergents have been recognized as the major preventive method against disease contraction and have been abundantly applied in hospital and domestic settings [102]. Acting as a non-specific selective pressure on the environmental microbiota, only resistant microorganisms are preserved. Between these, pathogens with antimicrobial drug resistance (AMR) represent an effective threat to human health. Hospital and, more generally, BE surfaces harbor many pathogenic-resistant microorganisms: various *Staphylococcus species* (among which the methicillin-resistant *S. aureus* is found) [83], members of the Enterobacteriaceae family (*E. coli*, for instance) that have beta-lactam, carbapenem, and colistin resistance genes, vancomycin-resistant *Enterococci*, and multidrug-resistant *Clostridium difficile*, *Acinetobacter* spp., and *Pseudomonas aeruginosa* [79]. To get rid of these persistent menaces, without, meanwhile, eradicating beneficial microbial species or promoting AMR (which are side effects of traditional chlorine-based detergents), the spread of selective sanitation procedures that also prevent recontamination is a crucial measure [84,85]. Meeting this necessity, detergents containing spores of *Bacillus* probiotics (Probiotic Cleaning Hygiene System, PCHS) have been shown to reduce up to 90% of surface HAI-associated microorganisms (such as *Staphylococcus* spp., *Enterobacteriaceae* spp., *Acinetobacter* spp., Mycetes, *Pseudomonas* spp., and *Clostridium* difficile), and, thus, HAI incidence due to a competitive exclusion mechanism between *Bacilli* and the other microorganisms [103,104,105,106]. In addition, PCHS contributes to a global reduction in microbiome resistance genes, counteracts fungal growth decreasing indoor air quality [107], and can inactivate harmful enveloped viruses (for instance, HCoV-229E and SARS-CoV-2 human coronaviruses, HSV-1, type A influenza viruses, and the modified Vaccinia virus Ankara) by means of enzymes able to process the components of viruses’ outer envelopes [105]. Despite the clear advances of these novel detergents, specific elimination of pathogenic species is achieved only by the addition of specific lytic bacteriophages to PCHS detergents, which also increases velocity and efficacy of the treatment [108]. PCHS represents a cheaper alternative to chemical disinfectants [87] and seems to be a feasible long-term sanitation strategy. Their safety has been indeed tested and, with the majority of *Bacillus* species being non-pathogenic for humans, probiotic-associated possible adverse events have been excluded [106]. Given this general overview, we believe that the diffusion of selective microbiome-manipulating detergents is one of the paths we can embark on to realize a renovated concept of hygiene, not only in hospitals, but in the complexity of the BE.

Again, the collaboration among project managers, medical staff, biologists, engineers, and architects is the key to designing a modern, healthier hospital.

## 7. Main Outcomes and Potential Impacts of an Urban Regeneration Based on Knowledge of the Microbiome of Built Environments

As emerges from a historical overview of urbanization, while science kept developing, little or no scientific discoveries were applied to shape and design a healthy BE, resulting in the rise in novel diseases of civilization. As scientists, we are responsible for the launch of a tight engagement of all the different stakeholders, including scientists, economists, psychologists, architects and engineers, policy makers, and, more importantly, civil society. It is difficult to perform health-promoting life activities when a huge lack of knowledge and awareness generally characterizes the overall population. The picture of the city of tomorrow is everything but idealistic. We indeed believe that, once the relevance and the impacts of a microbiome-inspired lifestyle are disseminated (together with all the related beneficial urban implications), the role of the society will be defined as an active contribution in determining health and the microbiome structure. The salubrious lifestyle of future citizens will be reflected by the structure of the BE in the course of renovation and vice versa. The main outcomes of a new holistic approach will range from the responsible use of cleaning products that protect microbiome diversity to the adoption of smart technologies to monitor the microbiome of the BE, from the improvement of contact with nature to the decrease in autoimmune and non-communicable diseases, from the promotion of MIGI constructions to the introduction of governance policies to ensure a healthier urban environment. These outcomes are reflected in the impacts on a wider perspective, as reported in Table 1.

## 8. Conclusions

After about one century since Le Corbusier’s visionary idea, we are still searching for the City of Tomorrow. However, what we have now is awareness about the complex and dynamic approach that is necessary to address this valuable aim.

Planning a city means taking into account, in a two-fold way, the health of the ecosystem as a whole (in which not only human beings are involved) and the multidimensional aspects of wellbeing, including social, cultural, and aesthetic values. In this, we are progressively accepting the invaluable role of microbial biodiversity and the ecosystem services that it could provide.

Only by integrating the different expertise of architects, botanists, economists, sociologists, and even microbiologists can the city of tomorrow be built from the ground up.

A special effort should be made for a city designed for the elderly and for children’s well-being and health: this social inclusion context can take advantage of the lesson learned from the microbial world, in which diversity and cooperation makes the difference.

## Figures and Tables

**Table 1:** Main outcomes and potential impacts of an urban regeneration based on knowledge of the microbiome of built environments.

Outcomes	Potential Impacts
Microbiome-Inspired Green Infrastructure	Regenerate nature Preserve and improve biodiversity
Greener cities with an increase contact with nature, healthier urban environment	Tackle autoimmune and non-communicable diseases
Smarter mass-transit systems	Limit the spread of AMR, healthier transit BE
Wise use of antimicrobial products, new approaches in buildings design	Limit the spread of AMR, introduce beneficial microbial communities
Smart technologies to monitor the microbiome of BE and to generate FAIR data	Microbiome perturbations prediction and prevention of disease outbreak
Stakeholders’ active engagement	Scientific innovations accepted
Introduction of governance policies to ensure a healthier urban environment	Scientific innovations implemented

## Data Availability

Not applicable.
